# Early Mycophenolate Mofetil Combination Therapy as an Effective Approach for Immune‐Related Hepatitis Induced by Immune Checkpoint Inhibitors in Patients With Solid Tumor

**DOI:** 10.1002/cam4.71720

**Published:** 2026-03-19

**Authors:** Yukiko Shimoda Igawa, Tatsuya Yoshida, Jun Sato, Yuri Yoshinami, Yukiko Hibino, Takamichi Arima, Yuta Maruki, Hirokazu Shoji, Kenjiro Namikawa, Kazuki Sudo, Yoshitaka Honma, Natsuko Okita, Hironobu Hashimoto, Naoya Yamazaki, Takuji Okusaka, Kan Yonemori, Noboru Yamamoto, Yuichiro Ohe, Ken Kato

**Affiliations:** ^1^ Department of Thoracic Oncology National Cancer Center Hospital Tokyo Japan; ^2^ Department of Experimental Therapeutics National Cancer Center Hospital Tokyo Japan; ^3^ Department of Gastrointestinal Medical Oncology National Cancer Center Hospital Tokyo Japan; ^4^ Department of Medical Oncology National Cancer Center Hospital Tokyo Japan; ^5^ Department of Pharmacy National Cancer Center Hospital Tokyo Japan; ^6^ Department of Hepatobiliary and Pancreatic Oncology National Cancer Center Hospital Tokyo Japan; ^7^ Department of Dermatologic Oncology National Cancer Center Hospital Tokyo Japan; ^8^ Department of Head and Neck, Esophageal Medical Oncology National Cancer Center Hospital Tokyo Japan

**Keywords:** hepatitis, immune checkpoint inhibitor, immune‐related adverse event, mycophenolate mofetil, solid tumor

## Abstract

**Background:**

Immune‐related hepatitis (ir‐hepatitis) is an immune‐related adverse event that can be resistant to systemic steroid therapy. Mycophenolate mofetil (MMF) is recommended for steroid‐refractory cases; however, evidence supporting its efficacy remains unclear. We aimed to evaluate the efficacy of MMF in patients with solid tumors, the optimal timing for administration, and its effect on cumulative steroid dosage.

**Methods:**

A retrospective cohort analysis was conducted between January 2015 and August 2023. We obtained data from eligible consecutive patients who developed ir‐hepatitis with grade ≥ 2 alanine aminotransferase (ALT) elevation requiring systemic steroids. Participants were divided into three groups: MMF‐early combination, MMF‐late combination, and systemic steroid‐only. ALT improvement rate was used to assess the efficacy of MMF based on the Common Terminology Criteria for Adverse Events version 5.0.

**Results:**

Among 4405 patients treated with immune checkpoint inhibitors, 151 (3.4%) developed ir‐hepatitis requiring systemic steroids, of whom 123 had grade ≥ 2 ALT elevation. The median patient age was 62 years (interquartile range: 54–72), and 42 patients (34%) received MMF. Forty‐one patients were evaluable for MMF timing. The ALT improvement rate on day 7 was significantly higher in the MMF‐early combination group (*n* = 10) than in the MMF‐late combination group (*n* = 31) (78.5% vs. 41.6%, *p* < 0.01). Among 40 patients evaluable for steroid dosage, cumulative systemic steroid dosage was significantly lower in the MMF‐early combination group (*n* = 8) than in the MMF‐late combination group (*n* = 32) (2121 mg vs. 3745 mg, *p* = 0.03). These effects were comparable even for the MMF‐early combination and systemic steroid‐only groups.

**Conclusions:**

Despite the small sample size, early combination therapy with MMF and systemic steroids rapidly improved ir‐hepatitis, consequently reducing the cumulative systemic steroid dosage.

## Graphical abstract

Our study highlights the benefits of early use of mycophenolate mofetil (MMF) for patients with immune‐related hepatitis requiring systemic steroids. This may provide guidance on the appropriate use of MMF in terms of its efficacy and safety.

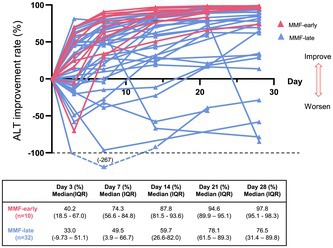

**Abbreviations**
ALT: alanine aminotransferase
AST: aspartate aminotransferase
CONSORT: Consolidated Standards of Reporting Trials
CTLA‐4: cytotoxic T‐lymphocyte antigen 4
ECOG: Eastern Cooperative Oncology Group
ICI: immune checkpoint inhibitor
IQR: interquartile range
ir: immune‐related
IrAE: immune‐related adverse event
Ir‐hepatitis: immune‐related hepatitis
MMF: mycophenolate mofetil
OS: overall survival
PD‐1: programmed cell death 1
PD‐L1: programmed death‐ligand 1
PS: performance status
T: biltotal bilirubin

## 1 Introduction

Immune checkpoint inhibitors (ICIs) have shown remarkable efficacy across a wide range of cancer types, expanding their use in recent years. However, ICIs can induce immune‐related adverse events (irAEs) owing to immune activation. The development of irAEs may be associated with the therapeutic efficacy of ICIs; however, severe irAEs requiring immunosuppression are associated with impaired progression‐free survival and overall survival (OS) [1].

Immune‐related hepatitis (ir‐hepatitis) is a type of irAEs. In phase III trials of the anti‐cytotoxic T‐lymphocyte antigen 4 (CTLA‐4) antibody, ipilimumab for malignant melanoma, the incidence of ir‐hepatitis of any grade was 1.2%–3.9%, with grade ≥ 3 occurring in 0%–1.6% of cases [2, 3, 4]. In phase III trials of the anti‐programmed cell death 1 (PD‐1) antibodies, nivolumab for malignant melanoma and non‐small cell lung cancer, and pembrolizumab for malignant melanoma, the incidence of ir‐hepatitis of any grade was 1.8%–6.0%, with grade ≥ 3 occurring in 0.3%–1.8% of cases [3, 4, 5, 6]. When anti‐CTLA‐4 and anti‐PD‐1/programmed death‐ligand 1 (PD‐L1) antibodies were used in combination, the incidence of ir‐hepatitis of any grade was 17.6%, with grade ≥ 3 occurring in approximately 8.3% of cases [4]. Meta‐analyses on irAEs have shown that among patients who developed fatal irAEs, ir‐hepatitis accounted for 16% of cases in the ipilimumab group, 22% in the anti‐PD‐1/PD‐L1 antibody group, and 22% in the combination therapy group. Fatal outcomes were reported in 10%–17% of ir‐hepatitis cases [7]. While relatively uncommon, severe ir‐hepatitis can therefore be life‐threatening.

The first‐line treatment for ir‐hepatitis involves systemic steroid administration. However, ir‐hepatitis occasionally results in resistance to systemic steroid treatment, necessitating additional immunosuppressive therapies. The management guidelines for ir‐hepatitis recommend combining mycophenolate mofetil (MMF) with systemic steroids for steroid‐refractory cases based on case reports and expert opinion [1, 8, 9, 10]; however, there has been no unified consensus on the treatment methods for MMF. In steroid‐refractory or steroid‐resistant cases, prolonged systemic steroid use can increase the risk of opportunistic infections, consequently worsening the prognosis [11, 12, 13]. A high dose of systemic steroids following ICI initiation can affect the efficacy of ICIs and the prognosis [14, 15]. However, data on whether the addition of MMF can resolve these issues are also lacking.

In this study, we evaluated the efficacy of MMF in patients with solid tumors and its optimal timing of administration to identify appropriate management strategies for ir‐hepatitis, as well as its effect on the cumulative steroid dosage required.

## 2 Materials and Methods

### 2.1 Study Population

We retrospectively reviewed the records of consecutive patients with solid tumors who developed ir‐hepatitis with grade ≥ 2 alanine aminotransferase (ALT) elevation requiring systemic steroids after ICI regimens (nivolumab, pembrolizumab, atezolizumab, avelumab, durvalumab, and ipilimumab) between January 2015 and August 2023 at the National Cancer Center Hospital, Tokyo, Japan. The following clinical data were evaluated: age, sex, Eastern Cooperative Oncology Group (ECOG)‐performance status (PS), cancer type, ICI regimen, hepatitis grade, concurrent cholangitis, and systemic steroid dose. The hepatitis grade was assessed at the start of the first treatment.

We divided the eligible patients into three groups: MMF‐early combination, MMF‐late combination, and systemic steroid‐only groups. The MMF‐early and MMF‐late combination groups were defined as MMF initiation within and after 3 days of the initial systemic steroid treatment, respectively. The 3‐day cutoff for defining early MMF initiation was predefined based on clinical practice guidelines [1, 8, 9, 10], which recommend considering MMF addition after 3 days of systemic steroid treatment in grade ≥ 2 steroid‐refractory cases. Given the lack of consensus regarding the optimal timing of MMF initiation, this cutoff was selected to assess the potential benefit of earlier MMF combination therapy. The systemic steroid‐only group was defined as patients treated with systemic steroids alone.

### 2.2 Assessment of Immune‐Related‐Hepatitis

Ir‐hepatitis was defined as an elevation in ALT, aspartate aminotransferase (AST), or total bilirubin levels in patients receiving regimens that include ICIs, in the absence of other causes of liver injury, such as viral hepatitis, ischemia, or toxicity of coexistent medications. AST, ALT, and total bilirubin levels were graded based on the Common Terminology Criteria for Adverse Events version 5.0 [16].

We evaluated changes in ALT levels as an indicator of MMF efficacy, in line with prior reports [17, 18, 19, 20]. ALT improvement rate (IR) was calculated as the ratio of the change in ALT from the initial treatment to a specific day, normalized by the ALT value at the initial treatment day. Improvement in ir‐hepatitis was defined as ALT grade ≤ 1, evaluated within 90 days after the start of the first treatment [21]. Ir‐hepatitis relapse was defined as a re‐increase in ALT grade ≥ 2 not attributable to other diseases other than ir‐hepatitis.

Steroid‐refractory was defined as the addition of MMF owing to initial non‐response, and steroid‐resistant was defined as the addition of MMF after initial response and inability to taper off systemic steroids [21]. The cumulative systemic steroid dosage was calculated as the sum of all systemic steroid administrations from the start of treatment until discontinuation, converted to prednisone equivalents. Systemic steroid tapering was performed in accordance with established clinical guidelines, with the timing and rate determined at the treating physician's discretion based on each patient's clinical condition and response.

Given that immune‐related cholangitis is rare but associated with poor prognosis, we also evaluated the impact of ir‐hepatitis driven by biliary injury on disease progression. Phenotype was evaluated using the classification for hepatitis and defined as meeting criteria for both cholestatic and mixed phenotypes [22, 23] (Table S1).

### 2.3 Statistical Analysis

Fisher's exact test was performed to assess differences in categorical data. Continuous variables between two groups were compared using the Mann–Whitney U test. Continuous variables among three groups were compared using one‐way analysis of variance or Welch's test when data were normally distributed, and the Kruskal–Wallis test when data were non‐normally distributed or ordinal.

The correlation between ALT IR and systemic steroid dosage was evaluated using Spearman's rank correlation coefficient. OS was measured from the first day of ir‐hepatitis onset to the day of death or the last follow‐up, excluding cases classified as Stages I–III. Survival outcomes were estimated using the Kaplan–Meier method and compared between groups using the log‐rank test.

All statistical analyses were performed using EZR version 1.54 [24] and GraphPad Prism 5.0 (GraphPad Software, San Diego, CA, USA). Statistical significance was set at a *p*‐value < 0.05.

Data were analyzed until patient death or until the data cutoff date (February 29, 2024), whichever was earliest.

### 2.4 Ethical Considerations

This study was approved by the Ethics Committee of the National Cancer Center Hospital (Tokyo, Japan) and was conducted in compliance with the Declaration of Helsinki (2023–153). All study participants provided informed consent.

## 3 Results

### 3.1 Participant Selection

**FIGURE 1:**
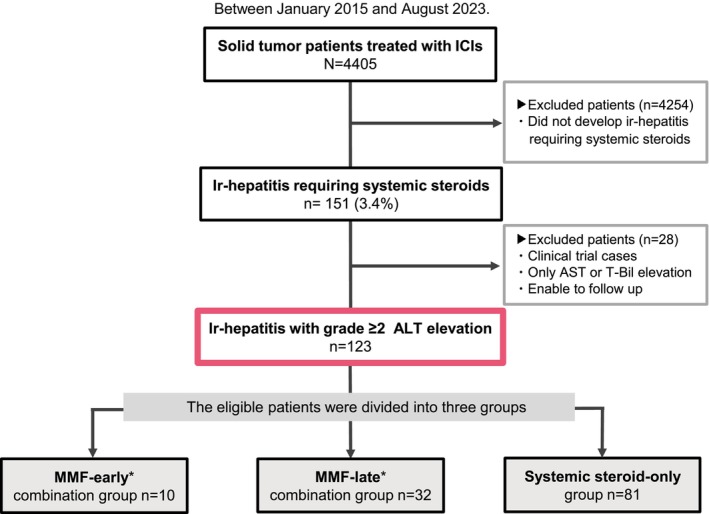
CONSORT diagram of the study. Eligible patients with ir‐hepatitis of grade ≥ 2 ALT elevation were divided into three groups: MMF‐early combination, MMF‐late combination, and systemic steroid‐only. The MMF‐early combination group was defined as MMF initiation within 3 days of initial systemic steroid treatment, and the MMF‐late combination group was defined as MMF initiation after 3 days of initial systemic steroid treatment.

Among 4405 patients treated with ICI‐containing regimens during the study period, 151 (3.4%) developed ir‐hepatitis requiring systemic steroids, including cases associated with biliary injury. Of these, 123 patients met the study inclusion criteria, defined as having grade ≥ 2 ALT elevation (Figure 1).

### 3.2 Patient Characteristics

The median patient age was 62 years (interquartile range [IQR]: 54–72), and 118 patients (96%) had an ECOG‐PS of 0/1. The cohort included 67 patients (55%) with melanoma, 31 (25%) with lung cancer, and 25 (20%) with other cancer types. The major regimens used were ICI + ICI (41%), ICI‐monotherapy (41%), and ICI + chemotherapy (18%) (Table 1).

**TABLE 1:** Patient baseline characteristics (*N* = 123).

Characteristics		Overall (*N* = 123)	MMF‐early (*N* = 10)	MMF‐late (*N* = 32)	Systemic steroid‐only (*N* = 81)	*p*
Age median, IQR, years		62 (54–72)	70 (66–71)	60 (50–68)	62 (55–73)	0.33
Sex, *n* (%)	Male	67 (54)	7 (70)	19 (59)	41 (51)	0.44
	Female	56 (46)	3 (30)	13 (41)	40 (49)	
ECOG‐PS, *n* (%)	0–1	118 (96)	9 (90)	32 (100)	77 (95)	0.30
	≤ 2	5 (4)	1 (10)	0	4 (5)	
Stage, *n* (%)	I–III	9 (7)	1 (10)	3 (9)	5 (6)	0.62
	IV/recurrence	114 (93)	9 (90)	29 (91)	76 (94)	
Cancer type, *n* (%)	Melanoma	67 (55)	0 (0)	13 (41)	54 (67)	< 0.01
	Lung cancer	31 (25)	3 (30)	14 (44)	14 (17)	
	Other cancer	25 (20)	7 (70)	5 (15)	13 (16)	
ICI regimen, *n* (%)	ICI‐monotherapy	50 (41)	3 (30)	15 (47)	32 (40)	0.23
	ICI + ICI	51 (41)	4 (40)	9 (28)	38 (47)	
	ICI + Chemotherapy	22 (18)	3 (30)	8 (25)	11 (13)	
Hepatitis grade, *n* (%)	2	33 (27)	1 (10)	8 (25)	24 (30)	0.04
	3	69 (56)	4 (40)	19 (59)	46 (57)	
	4	21 (17)	5 (50)	5 (16)	11 (13)	
Hepatitis driven by biliary injury^a^	Yes	15 (12)	2 (20)	9 (28)	4 (5)	< 0.01
	No	108 (88)	8 (80)	23 (72)	77 (95)	
Initial steroid^b^ dosage^c^, median, IQR, mg		50 (30–68)	65 (53–137)	50 (30–70)	50 (30–60)	0.36
Steroid^b^ response^d^	Refractory	NA	10 (100)	9 (28)	NA	< 0.01
	Resistance		0 (0)	23 (72)		
Duration from steroid^b^ initiation to MMF, median, IQR, days		NA	2 (1–2)	16 (6–42)	NA	< 0.01

Abbreviations: ECOG, Eastern Cooperative Oncology Group; ICI, immune‐checkpoint inhibitor; IQR, Interquartile Range; MMF, mycophenolate mofetil; NA, not applicable; PS, performance status.

^a^ Classified as cholestasis or mixed type of hepatitis.

^b^ Steroid means systemic steroid.

^c^ Calculated in prednisolone equivalents.

^d^ Definition of steroid response: refractory, addition of MMF owing to initial nonresponse; resistance, addition of MMF after initial response and inability to taper off systemic steroids.

The 123 patients who developed grade ≥ 2 ALT elevation were divided into three groups based on the ir‐hepatitis treatment strategy: MMF‐early combination (*n* = 10), MMF‐late combination (*n* = 32), and systemic steroid‐only (*n* = 81) (Figure 1). In the MMF combination group, all patients received MMF at 2000 mg/day; two started at 1000 mg with subsequent increase. The median duration from the start of systemic steroid treatment to MMF administration was 9 days (IQR: 4–28), including 2 days (IQR: 1–2) in the MMF‐early group and 16 days (IQR: 6–42) in the MMF‐late group. All patients in the MMF‐early combination group were steroid‐refractory (10/10, 100%), whereas most patients (23/32, 72%) in the MMF‐late combination group were steroid‐resistant. The median duration of MMF use until improvement to grade ≤ 1 was 84 days (IQR: 9–299).

Regarding baseline characteristics, there were no significant differences in age, sex, or ECOG‐PS among the three groups. However, the MMF‐early group had a higher proportion of patients with severe ir‐hepatitis (grade 4) than did the other groups, accounting for 50% of cases (5/10). The proportion of ir‐hepatitis associated with biliary injury was higher in the MMF‐treated groups (MMF‐early and ‐late combination groups) than in the systemic steroid‐only group (26% [11/42] vs. 5% [4/81], respectively) (Table 1).

### 3.3 Efficacy of Early Mycophenolate Mofetil for Immune‐Related Hepatitis

**FIGURE 2:**
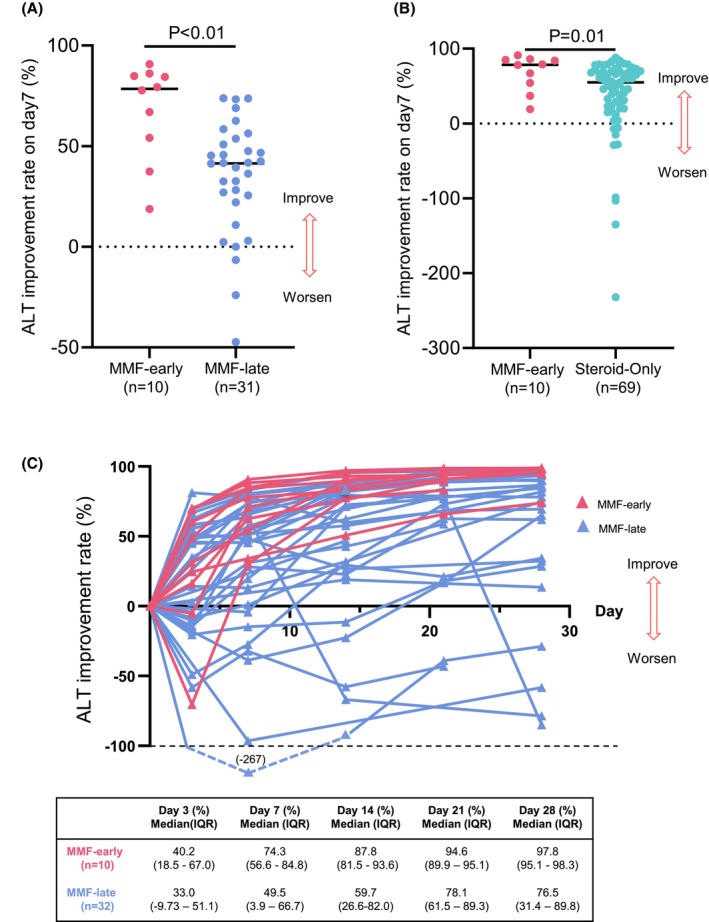
Efficacy of early MMF for ir‐hepatitis. (A) The difference in ALT IR on day 7 resulting from MMF treatment between the MMF‐early and MMF‐late combination groups (median: 78.5% vs. 41.6%). (B) The difference in ALT IR on day 7 between the MMF‐early combination group after MMF treatment and the systemic steroid‐only group after systemic steroid treatment (median: 78.5% vs. 54.8%). (C) Course of ALT IR up to 28 days after the start of the initial treatment. Evaluable patients were assessed on days 3, 7, 14, 21, and 28. ALT, alanine aminotransferase; IQR, interquartile range; IR, improvement rate; ir, immune‐related; MMF, mycophenolate mofetil.

Overall, 42 of 123 patients (34%) received MMF. The median duration from initiation of systemic steroid treatment to improvement was 19 days (IQR: 10–36); this duration was significantly longer in the MMF‐late combination group (median: 39 days; IQR: 31–70) (Table 2). Among the 41 patients evaluable for the impact of MMF timing on the change in ir‐hepatitis, the ALT IR resulting from MMF treatment on day 7 in the MMF‐early combination group (*n* = 10) was significantly higher than that in the MMF‐late combination group (*n* = 31) (78.5% vs. 41.6%, *p* < 0.01) (Figure 2A). The ALT IR on day 7 after MMF treatment in the MMF‐early combination group was also significantly higher than that on day 7 after systemic steroid treatment in the systemic steroid‐only group (*n* = 69) (78.5% vs. 51.9%, *p* = 0.01) (Figure 2B). Figure 2C shows the course of ALT IR up to 28 days after the start of the initial treatment. Data points were assessed on days 3, 7, 14, 21, and 28. The MMF‐early combination group showed rapid improvement after day 7, whereas the MMF‐late combination group included cases with slow improvement or even deterioration.

**TABLE 2:** Efficacy of MMF on the time to improvement and relapse rate (*N* = 123).

Outcome	Overall (*N* = 123)	MMF‐early (*N* = 10)	MMF‐late (*N* = 32)	Systemic steroid‐only (*N* = 81)	*p*
Duration from treatment initiation to improvement, median, IQR, days	19 (10–36)	15 (10–20)	39 (31–70)	16 (9–28)	< 0.01
Improvement^a^ within 90 days, (%)	93/102 (91)	10/10 (100)	16/21 (76)	67/71 (94)	0.04
Relapse^b^ rate, (%)	39/118 (33)	0/9 (0)	16/30 (53)	23/79 (29)	< 0.01

*Note:* Since only evaluable patients were included, there was a discrepancy in the number of patients analyzed for both improvement within 90 days and relapse rate.

Abbreviations: IQR, Interquartile Range; MMF, mycophenolate mofetil.

^a^ Improvement was defined as ALT becoming grade ≤ 1 (Excluding cases of exacerbation).

^b^ Relapse was defined as a reincrease in ALT grade ≥ 2 not attributable to any diseases other than ir‐hepatitis.

The efficacy of MMF on time to improvement and relapse rate is summarized in Table 2. Because evaluation of improvement and relapse required laboratory data obtained at specific time points, analyses were limited to patients with appropriately timed laboratory measurements, resulting in differences in the number of evaluable cases. Overall, 91% of patients showed improvement within 90 days of initiating systemic steroid therapy. All patients with ir‐hepatitis in the MMF‐early combination group showed improvement within 90 days. The MMF‐early combination group had a significantly higher IR within 90 days than did the other groups (IR within 90 days: 100% vs. 76% vs. 94%, *p* = 0.04). There was also a significant difference in the relapse rate among the three groups, with the relapse rate being particularly worse in the MMF‐late combination group (relapse rate: 0% vs. 53% vs. 29%, *p* < 0.01) (Table 2).

### 3.4 Steroid Use During Mycophenolate Mofetil Treatment

**FIGURE 3:**
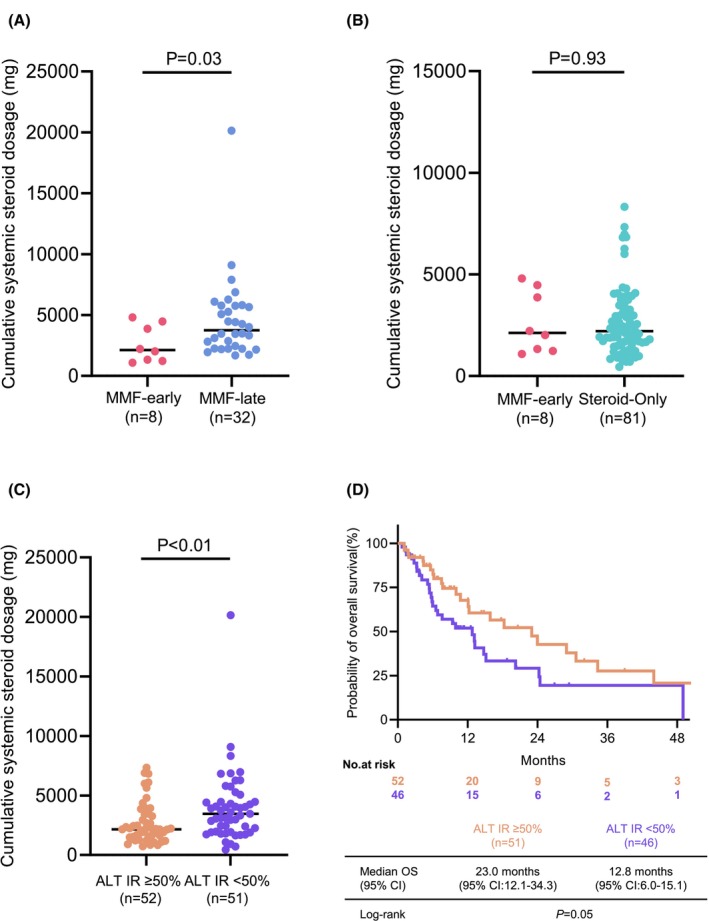
Impact of early MMF on steroid dosage and association of early improvement in ir‐hepatitis with prognosis. (A) The difference in required systemic steroid dosage between the MMF‐early and MMF‐late combination groups (median: 2121 mg vs. 3745 mg). (B) The difference in required systemic steroid dosage between the MMF‐early combination and systemic steroid‐only groups (median: 2121 mg vs. 2210 mg). (C) The difference in required systemic steroid dosage between patients with ALT IR on day 7 ≥ 50% and those with ALT IR < 50% (median: 2160 mg vs. 3470 mg). (D) Kaplan–Meier curves of OS for patients with ALT IR ≥ 50% on day 7 and those with ALT IR < 50% excluding cases classified as Stage I–III (median: 23.0 months vs. 12.8 months). ALT, alanine aminotransferase; IR, improvement rate; ir, immune‐related; MMF, mycophenolate mofetil; OS, overall survival.

Among the 40 patients evaluable for cumulative systemic steroid dosage during ir‐hepatitis, two patients in the MMF‐early combination group were excluded because they were transferred to other hospitals during the clinical course and could not be followed longitudinally. The cumulative systemic steroid dosage was significantly lower in the MMF‐early combination group (*n* = 8) than in the MMF‐late combination group (*n* = 32) (median: 2121 mg vs. 3745 mg, *p* = 0.03) (Figure 3A). Conversely, the cumulative systemic steroid dosage in the MMF‐early combination group (*n* = 8) did not exceed that of the systemic steroid‐only group (*n* = 81) (median: 2121 mg vs. 2210 mg, *p* = 0.93) (Figure 3B), although the MMF‐early combination group had more severe ir‐hepatitis (Table 1).

Regarding the correlation between ALT IR on day 7 and the required systemic steroid dosage, the required systemic steroid dosage tended to increase as ALT IR worsened (Figure S1).

### 3.5 Impact of Early Improvement in iImmune‐Related‐Hepatitis on Prognosis

Figure 3C,D show the impact of early ALT IR on cumulative systemic steroid dosage and OS, respectively. When patients with evaluable data were stratified according to ALT IR on day 7 (≥ 50% vs. < 50%), those with ALT IR ≥ 50% required significantly lower cumulative systemic steroid dosages (median: 2160 mg vs. 3470 mg, *p* < 0.01) (Figure 3C) and demonstrated longer OS (median: 23.0 months vs. 12.8 months, *p* = 0.05) (Figure 3D).

### 3.6 Effect of Cholangitis Complications on Systemic Steroid Use and Overall Survival

Additionally, we evaluated the effect of cholangitis complications on systemic steroid dosage and OS. Patients with cholangitis complications required significantly higher cumulative systemic steroid dosages (median: 3758 mg vs. 2350 mg, *p* < 0.01) (Figure S2A) and had worse OS (median: 5.7 months vs. 20.2 months, *p* = 0.05) (Figure S2B) than did those without cholangitis complications.

## 4 Discussion

This study showed that early combination therapy with MMF and systemic steroids could rapidly improve ir‐hepatitis, reducing cumulative systemic steroid dosage. Additionally, a higher rate of early improvement in liver function appeared to be associated with more favorable OS. These data suggest clinically relevant benefits of the early MMF combination therapy with systemic steroids for ir‐hepatitis, particularly for achieving early improvement in liver dysfunction. These emphasize caution when using MMF in patients with ir‐hepatitis requiring steroids.

MMF, (E)‐6‐(1,3‐dihydro‐4‐hydroxy‐6‐methoxy‐7‐methyl‐3‐oxo‐5‐isobenzofuranyl)‐4‐methylhexenoic acid 2‐(4‐morpholinyl) ethyl ester, is a prodrug of mycophenolic acid. Mycophenolic acid exerts its immunosuppressive effect by inhibiting purine metabolism, thereby inhibiting the synthesis of activated T and B lymphocytes [25, 26, 27]. MMF has been approved for the suppression of rejection in various organ transplants (kidney, heart, liver, lung, pancreas, and hematopoietic stem cell transplantation) and in nephrotic syndrome.

In current management guidelines for irAEs [1, 8, 9, 10], combined MMF and systemic steroids are recommended in steroid‐refractory or steroid‐resistant cases. However, the efficacy of MMF for ir‐hepatitis is based on a case series, and no evidence has been presented on the timing of MMF initiation or its use in steroid‐refractory or steroid‐resistant cases. The present study showed that using MMF within 3 days of the initial systemic steroid treatment significantly improved ir‐hepatitis, which also reduced the cumulative systemic steroid dosage and suppressed relapse. Similar findings have been reported in previous studies of patients with immune‐related colitis, another irAE, in which early combination therapy with immunosuppressants and short‐term systemic steroids shortened time to symptom improvement and was associated with significantly fewer complications from immunosuppression than those in patients treated with systemic steroids only [28, 29]. Systemic steroids provide clinical benefits for managing irAEs; however, the impact of long‐term systemic steroid use on systemic steroid complications and prognosis is a concern [30]. These findings support the early use of combined immunosuppressants for irAEs requiring systemic steroids, aligning with our results.

Regarding the mechanisms underlying irAE, Hellmann et al. proposed four concepts: increasing T‐cell activity against antigens present in both tumors and healthy tissues, increasing levels of pre‐existing autoantibodies, increasing levels of inflammatory cytokines, and enhanced complement‐mediated inflammation resulting from the direct binding of an antibody against CTLA‐4 expressed on normal tissues, such as the pituitary gland [31]. Additional mechanisms continue to be elucidated [32]. Evidence from research in patients with melanoma could support the concept that T cells involved in irAEs are clonally related to the cells primarily mediating an anti‐tumor response [33]. Karijn et al. hypothesized that an early combination therapy with immunosuppressants could be supported by the mechanism of clone migration, either from regional tumor‐draining lymph nodes or remote priming. The former suggests that biologics, ideally targeting lymphocyte homing, could limit T‐cell trafficking to tumors while treating irAEs [32]. Ir‐hepatitis is generally considered to resemble autoimmune‐type acute hepatitis with panlobular active hepatitis and inflammatory infiltrates, such as immune‐cell infiltration, including CD8+ T lymphocytes [34, 35, 36]. The increased effectiveness of early combination therapy with MMF observed in the present study may reflect suppression of lymphocyte dispersion, as suggested by Karijn et al. Additionally, because it takes a couple of days for MMF blood concentrations to reach a steady state [37, 38], an initial immunological event can cause sufficient damage, making subsequent immunosuppression too late to effectively treat the condition.

This study had some limitations. First, this was a retrospective study conducted at a single comprehensive cancer center with a limited sample size, particularly in the MMF‐early combination group (*n* = 10). MMF use was based on case severity, which may introduce a selection bias. Notably, despite more severe baseline hepatic dysfunction, likely reflecting this selection bias, the MMF‐early combination group demonstrated more favorable outcomes than did the MMF‐late combination and systemic steroid‐only groups, which included a higher proportion of mild cases. Additionally, interpretation of the marked difference in relapse rates between the MMF‐early (0%, 0/9 evaluable patients) and MMF‐late (53%, 16/30 evaluable patients) combination groups requires caution, as this finding is highly susceptible to the effects of small sample size. Despite these limitations, these findings suggest that early MMF combination therapy may be beneficial, particularly in patients with severe ir‐hepatitis requiring systemic steroids. Second, we evaluated ir‐hepatitis based on ALT values, which do not cover all types of ir‐hepatitis because ir‐hepatitis occasionally complicates cholangitis. Our study included only two patients with cholangitis complications in the MMF‐early combination group; however, the observed outcomes suggest potential benefits of early MMF combination therapy for such patients. Third, we did not conduct immune monitoring to explain how early MMF combination therapy rapidly improves ir‐hepatitis compared with late MMF combination therapy or treatment with systemic steroids alone. Further studies on the immunological effects of additional MMF treatment on ir‐hepatitis are warranted [39].

## 5 Conclusions

Early combination therapy with MMF and systemic steroids was associated with a rapid improvement of ir‐hepatitis and a reduction in the cumulative systemic steroid dosage. MMF should be considered for early combination use in severe ir‐hepatitis or in ir‐hepatitis cases requiring systemic steroids, considering the possibility of steroid resistance. To provide additional data on the contributing risk factors for ir‐hepatitis, including cholangitis complications, and to support predictive biomarker development and the establishment of standardized protocols, a phase III trial is currently underway to evaluate the efficacy of upfront steroid treatment with MMF for ir‐hepatitis in patients with solid tumors treated with ICIs.

## Author Contributions

**Yukiko Shimoda Igawa:** conceptualization (equal), data curation (lead), formal analysis (lead), investigation (lead), methodology (equal), visualization (lead), writing – original draft (lead). **Tatsuya Yoshida:** conceptualization (equal), methodology (equal), supervision (equal), visualization (equal), writing – review and editing (equal). **Jun Sato:** writing – review and editing (equal). **Yuri Yoshinami:** writing – review and editing (equal). **Yukiko Hibino:** writing – review and editing (equal). **Takamichi Arima:** writing – review and editing (equal). **Yuta Maruki:** writing – review and editing (equal). **Hirokazu Shoji:** writing – review and editing (equal). **Kenjiro Namikawa:** writing – review and editing (equal). **Kazuki Sudo:** writing – review and editing (equal). **Yoshitaka Honma:** writing – review and editing (equal). **Natsuko Okita:** writing – review and editing (equal). **Hironobu Hashimoto:** writing – review and editing (equal). **Naoya Yamazaki:** writing – review and editing (equal). **Takuji Okusaka:** writing – review and editing (equal). **Kan Yonemori:** writing – review and editing (equal). **Noboru Yamamoto:** writing – review and editing (equal). **Yuichiro Ohe:** writing – review and editing (equal). **Ken Kato:** supervision (equal), writing – review and editing (equal).

## Funding

This study did not receive any specific grants from funding agencies in the public, commercial, or non‐profit sectors.

## Disclosure

No other disclosures were reported.

## Ethics Statement

The study design was approved by the Ethics Committee of the National Cancer Center Hospital, Tokyo, Japan (2023‐153) and was conducted in compliance with the Declaration of Helsinki. Informed consent was obtained from all patients.

## Conflicts of Interest

Tatsuya Yoshida reports research funding from Abbvie, Amgen, AstraZeneca, Bristol‐Myers Squibb, Chugai/Roche, Daiichi Sankyo, Merck Sharp & Dohme (MSD), Novartis, Ono Pharmaceutical, and Takeda Pharmaceutical; honoraria from Amgen, AstraZeneca, Bayer, Bristol‐Myers Squibb Japan, Chugai/Roche, Daiichi Sankyo/UCB Japan, Eli Lilly Japan, MSD Oncology, Nippon Boehringer Ingelheim, Novartis, Ono Pharmaceutical, Pfizer, and Takeda Pharmaceutical; consulting or advisory roles for Amgen, AstraZeneca, Boehringer Ingelheim, Chugai/Roche, Eli Lilly Japan, and MSD. Jun Sato reports honoraria from Chugai Pharmaceutical, Eisai, and Fuji Film Pharma. Hirokazu Shoji reports research funding from Chugai Pharmaceutical, Daiichi Sankyo, Ono Pharmaceutical, and Takeda Pharmaceutical; consulting or advisory roles for Astellas Pharma, Ono Pharmaceutical, and Zymeworks. Kenjiro Namikawa reports honoraria from Bristol‐Myers Squibb Japan, MSD, Novartis, and Ono Pharmaceutical; consulting or advisory roles for MSD, and Novartis. Kazuki Sudo reports research funding from AstraZeneca, Boehringer Ingelheim, Chugai Pharmaceutical, Daiichi Sankyo, Eisai, Eli Lilly Japan, Genmab, Haihe, Kyowa Hakko Kirin, Merk Biopharma, MSD, Nihon Kayaku, Novartis, Ono Pharmaceutical, Pfizer, Sanofi, Seattle Genetics, Taiho, and Takeda Pharmaceutical; honoraria from Astellas Pharma, AstraZeneca, Bayer, Boehringer Ingelheim, Bristol‐Myers Squibb Japan, Chugai, Daiichi Sankyo, Eisai, Eli Lilly Japan, Fuji Film Pharma, Jansen, Merk Biopharma, MSD, Novartis, Ono Pharmaceutical, PDR Pharma, Pfizer, Sanofi, and Takeda Pharmaceutical; consulting or advisory roles for AstraZeneca, Eisai, Genmab, Gilead, Henlius, MSD, Novartis, OncXerna, Sanofi, and Takeda Pharmaceutical. Yoshitaka Honma reports research funding from Adlai Nortye Biopharma, Astellas Pharma, AstraZeneca, Chugai Pharmaceutical, Genmab, GlaxoSmithKline (GSK), Janssen, Maruho, Merck Biopharma, MSD, Rakuten Medical Japan, and Taiho Pharmaceutical; honoraria from Bayer, Bristol Myers Squibb Japan, Chugai Pharmaceutical, Eisai, Eli Lilly Japan, Merck Biopharma, MSD, Novartis, Nutri, Ono Pharmaceutical, Taiho Pharmaceutical, and Teijin Pharma; consulting or advisory roles for Janssen, and Rakuten Medical Japan. Hironobu Hashimoto reports honoraria from Chugai Pharmaceutical, Shionogi, and Taiho Pharmaceutical. Naoya Yamazaki reports honoraria from Bristol‐Myers Squibb, GSK, and Novartis Pharma; royalties or licenses from Bristol‐Myers Squibb, Ono Pharmaceutical, and Takeda Pharmaceutical. Takuji Okusaka reports research funding from AstraZeneca, Bristol‐Myers Squibb Japan, Chiome Bioscience, Chugai Pharmaceutical, Eisai, Incyte Japan, MSD, Novartis Pharma, Syneos, and SYSMEX; honoraria from AstraZeneca, Chugai Pharmaceutical, Daiichi Sankyo, Eisai, Johnson & Johnson, Myriad Genetics, Nihon Servier, Novartis Pharma, Ono Pharmaceutical, Syneos, Taiho Pharmaceutical, and Yakult Honsha; consulting or advisory roles for AstraZeneca, Chugai Pharmaceutical, Daiichi Sankyo, Sumitomo Pharma, Eisai, FUJIFILM Toyama Chemical, Nihon Servier, Novartis Pharma, and Ono Pharmaceutical. Takuji Okusaka is also an editorial board member of Cancer Science. Kan Yonemori reports research funding from AstraZeneca, Boehringer Ingelheim, Chugai Pharmaceutical, Daiichi Sankyo, Eisai, Eli Lilly Japan, Genmab, Haihe, Kyowa Hakko Kirin, Merk Biophama, MSD, Nihon Kayaku, Novartis, Ono Pharmaceutical, Pfizer, Sanofi, Seattle Genetics, Taiho, and Takeda Pharmaceutical; honoraria from Astellas Pharma, AstraZeneca, Bayer, Boehringer Ingelheim, Bristol‐Myers Squibb Japan, Chugai, Daiichi Sankyo, Eisai, Eli Lilly Japan, Fuji Film Pharma, Jansen, Merk Biopharma, MSD, Novartis, Ono Pharmaceutical, PDR Pharma, Pfizer, Sanofi, and Takeda Pharmaceutical; consulting or advisory roles for AstraZeneca, Eisai, Genmab, Gilead, Henlius, MSD, Novartis, OncXerna, Sanofi, and Takeda Pharmaceutical. Noboru Yamamoto reports research funding from Abbvie, Amgen, Astellas Pharma, AstraZeneca, Bayer, Bicycle Therapeutics, Boehringer Ingelheim, Bristol‐Myers Squibb, Carna Biosciences, Chiome Bioscience, Chugai Pharma, CMIC, Daiichi Sankyo, Eisai, Eli Lilly Japan, Genmab, GSK, InventisBio, Janssen, Kaken Pharmaceutical, Kyowa Kirin, Merck Serono, MSD, Novartis, Ono Pharmaceutical, Otsuka, Pfizer, Rakuten Medical, Shionogi, Sumitomo Dainippon Pharma, Taiho Pharmaceutical, Takeda, and Toray Industries; honoraria from Chugai Pharma, Daiichi Sankyo, Eisai; consulting or advisory roles for Boehringer Ingelheim, Chugai Pharma, CMIC, Eisai, Healios, Merck, Noile‐Immune Biotech, and Takeda Pharmaceutical. Yuichiro Ohe reports research funding from AstraZeneca, Bristol‐Myers Squibb Japan, Chugai Pharma, Eli Lilly Japan, Janssen, Novartis, Ono Pharmaceutical, Pfizer, Taiho Pharmaceutical, and Takeda Pharmaceutical; receiving honoraria from Amgen, AstraZeneca, Bayer, Boehringer Ingelheim, Bristol‐Myers Squibb Japan, Chugai Pharma, Eisai, Eli Lilly Japan, Kyowa Kirin, MSD, Nippon Kayaku, Novartis, Ono Pharmaceutical, Pfizer, Taiho Pharmaceutical, and Takeda Pharmaceutical; consulting or advisory roles for Amgen, Anheart Therapeutics, AstraZeneca, Celltrion, Chugai Pharma, Eli Lilly Japan, Novartis, Ono Pharmaceutical, PharmaMar, and Takeda Pharmaceutical. Ken Kato reports research funding from AstraZeneca, Bayer, BeiGene, Bristol‐Myers Squibb Japan, Chugai Pharma, MSD, Ono Pharmaceutical, Shionogi; receiving honoraria from Bristol‐Myers Squibb Japan, Ono Pharmaceutical, and Taiho Pharmaceutical; consulting or advisory roles for AstraZeneca, Bayer, BeiGene, Bristol‐Myers Squibb Japan, MSD, and Roche.

## Supporting information


**Appendix S1:** cam471720‐sup‐0001‐AppendixS1.pdf. **Figure S1:** Correlation between ALT IR and required steroid dosage.
**Figure S2:** Clinical outcomes of ir‐hepatitis with cholangitis. (A) Difference in the required systemic steroid dosage between patients with hepatitis with and without cholangitis (median: 3758 mg vs. 2350 mg). (B) Kaplan–Meier curves of OS in patients with and without cholangitis excluding cases classified as Stage I–III (median: 5.7 months vs. 20.2 months).
**Table S1:** Definition of classification for hepatitis.

## Data Availability

The data that supports the findings of this study are available from the corresponding author upon reasonable request.
