# Supplementary material for: Early Mycophenolate Mofetil Combination Therapy as an Effective Approach for Immune‐Related Hepatitis Induced by Immune Checkpoint Inhibitors in Patients With Solid Tumor
Source: Cancer Med. 2026 Mar 19;15(3):e71720. doi: 10.1002/cam4.71720 (PMC13093305; doi:10.1002/cam4.71720)
Supplement: Supplementary file 1 — Appendix S1: cam471720‐sup‐0001‐AppendixS1.pdf. Figure S1: Correlation between ALT IR and required steroid dosage. Figure S2: Clinical outcomes of ir‐hepatitis with cholangitis. (A) Difference in the required systemic steroid dosage between patients with hepatitis with and without cholangitis (median: 3758 mg vs. 2350 mg). (B) Kaplan–Meier curves of OS in patients with and without cholangitis excluding cases classified as Stage I–III (median: 5.7 months vs. 20.2 months). Table S1: Definition of classification for hepatitis. [file CAM4-15-e71720-s001.pdf]

Table S1. Definition of classification for hepatitis

**Classification for hepatitis**	**Definition**
Hepatocellular injury phenotype	2ULN < ALT + ALP ≤ ULN or 5 ≤ ALT/ ALP
Cholestatic phenotype	ALT ≤ ULN + 2ULN < ALP or ALT/ ALP ≤ 2
Mixed phenotype	2ULN < ALT + ULN < ALP and 2 < ALT/ ALP < 5

Abbreviations: ALP, alkaline phosphatase; ALT, alanine aminotransferase; ULN, upper limit of normal

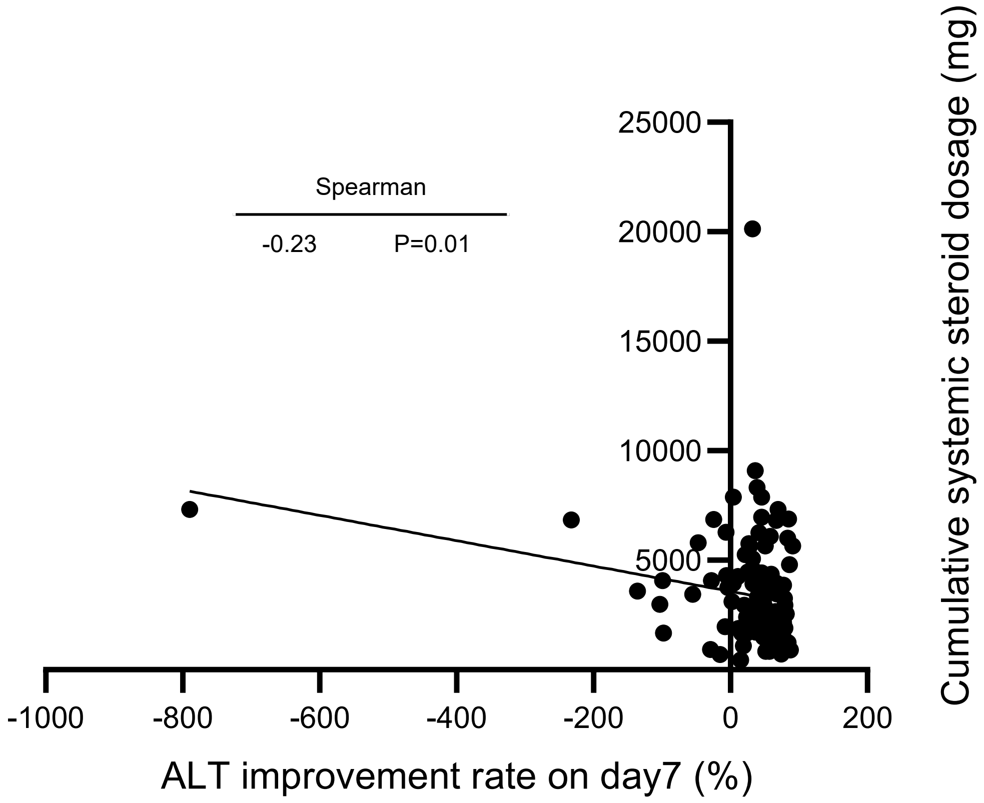

Figure S1. Correlation between ALT IR and required steroid dosage
The correlation between ALT IR and systemic steroid dosage was evaluated using Spearman's rank correlation coefficient.

Abbreviations: ALT, alanine aminotransferase; IR, improvement rate

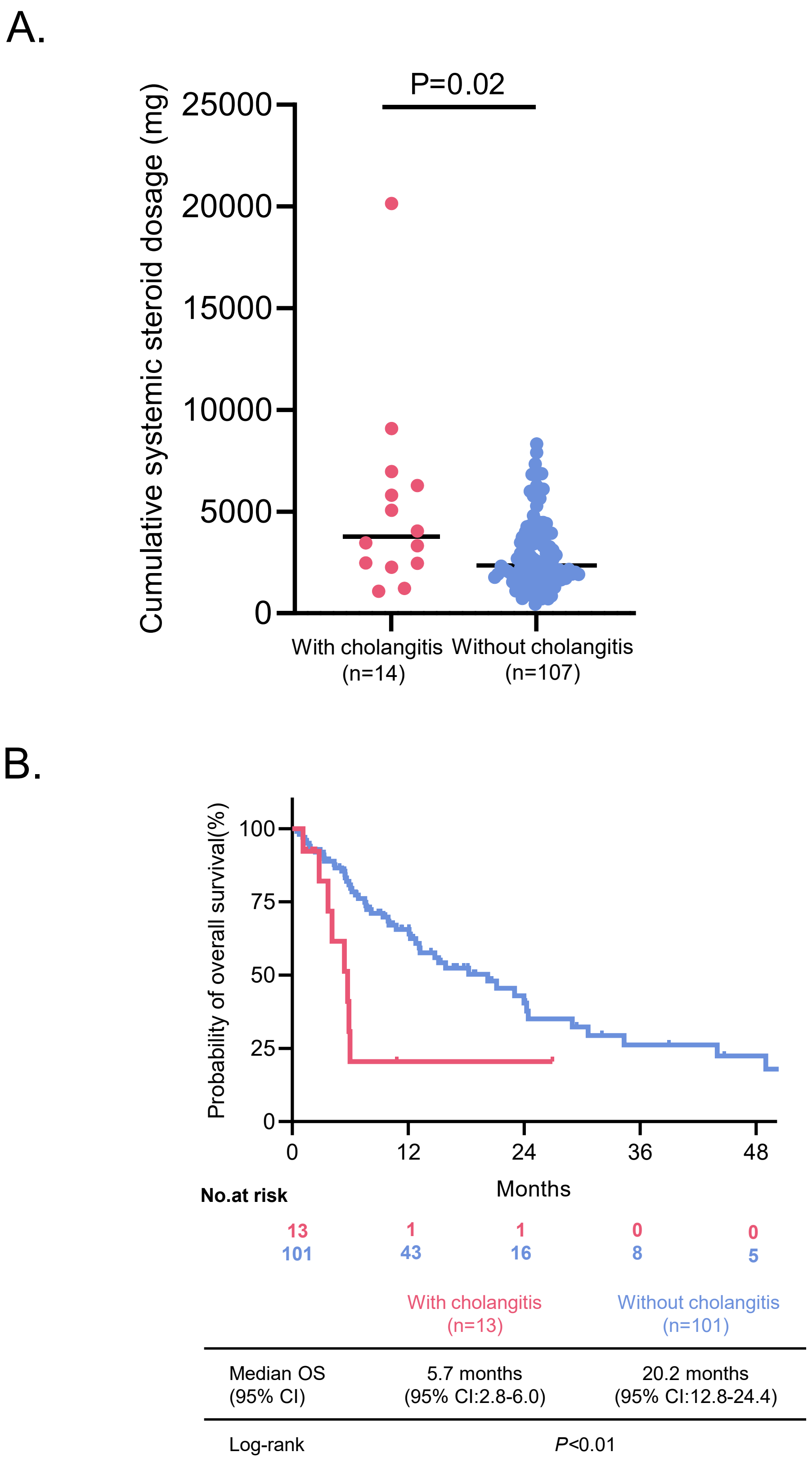

Figure S2. Clinical outcomes of ir-hepatitis with cholangitis

(A) Difference in the required systemic steroid dosage between patients with hepatitis with and without cholangitis (median: 3758 mg vs. 2350 mg).

(B) Kaplan–Meier curves of OS in patients with and without cholangitis excluding cases classified as Stage I–III (median: 5.7 months vs. 20.2 months).

Abbreviations: ir, immune-related; CI, confidence interval; OS, overall survival
